# Predictors of RSV LRTI Hospitalization in Infants Born at 33 to 35 Weeks Gestational Age: A Large Multinational Study (PONI)

**DOI:** 10.1371/journal.pone.0157446

**Published:** 2016-06-16

**Authors:** Zbyněk Straňák, Elie Saliba, Paraskevi Kosma, Klara Posfay-Barbe, Khalid Yunis, Teresa Farstad, Kristina Unnebrink, Jean van Wyk, Colleen Wegzyn, Gerard Notario, Stefanie Kalus, Fiona J. Campbell

**Affiliations:** 1 Institute for the Care of Mother and Child, Third Faculty of Medicine, Charles University, Prague, Czech Republic; 2 Inserm U930, Université François Rabelais, and Department of Neonatology, University Hospital Clocheville, Tours, France; 3 Department of Woman and Child Health, Karolinska Institutet, Stockholm, Sweden; 4 Department of Pediatrics, Geneva Medical School and University Hospitals of Geneva, Geneva, Switzerland; 5 Department of Pediatrics and Adolescent Medicine, American University of Beirut, Riad El Solh, Beirut, Lebanon; 6 Department of Pediatrics, Akershus University Hospital, Lørenskog, Norway; 7 Data and Statistical Sciences, AbbVie Deutschland GmbH & Co. KG, Ludwigshafen, Germany; 8 Virology, AbbVie Inc., North Chicago, IL, United States of America; 9 Neonatology, AbbVie Inc., North Chicago, IL, United States of America; 10 Biostatistics, GKM Gesellschaft für Therapieforschung mbH, Munich, Germany; 11 Neonatology and HIV, AbbVie Ltd, Dublin, Ireland; University of North Carolina at Chapel Hill, UNITED STATES

## Abstract

**Background:**

Preterm infants are at high risk of developing respiratory syncytial virus (RSV)-associated lower respiratory tract infection (LRTI). This observational epidemiologic study evaluated RSV disease burden and risk factors for RSV-associated LRTI hospitalization in preterm infants 33 weeks+0 days to 35 weeks+6 days gestational age not receiving RSV prophylaxis.

**Methods:**

Preterm infants ≤6 months of age during RSV season (1 October 2013–30 April 2014) were followed at 72 sites across 23 countries from September 2013–July 2014 (study period). RSV testing was performed according to local clinical practice. Factors related to RSV-associated hospitalization for LRTI were identified using multivariable logistic regression with backward selection.

**Results:**

Of the 2390 evaluable infants, 204 and 127 were hospitalized for LRTI during the study period and RSV season, respectively. Among these subjects, 64/204 and 46/127, respectively, were hospitalized for confirmed RSV LRTI. Study period and RSV season normalized RSV hospitalization rates (per 100 infant years) were 4.1 and 6.1, respectively. Factors associated with an increased risk of RSV-related LRTI hospitalization in multivariable analyses were smoking of family members (*P*<0.0001), non-hemodynamically significant congenital heart disease diagnosis (*P* = 0.0077), maternal age of ≤25 years at delivery (*P* = 0.0009), low maternal educational level (*P* = 0.0426), household presence of children aged 4 to 5 years (*P* = 0.0038), age on 1 October ≤3 months (*P* = 0.0422), and presence of paternal atopy (*P*<0.0001).

**Conclusions:**

During the 2013–2014 RSV season across 23 countries, for preterm infants 33–35 weeks gestation ≤6 months old on 1 October not receiving RSV prophylaxis, confirmed RSV LRTI hospitalization incidence was 4.1 per 100 infant years during the study period and 6.1 per 100 infant years during the RSV season. This study enhances the findings of single-country studies of common risk factors for severe RSV infection in preterm infants and suggests that combinations of 4–6 risk factors may be used to accurately predict risk of RSV hospitalization. These findings may be useful in the identification of infants most at risk of severe RSV infection.

## Introduction

By the age of 2, most children will experience a respiratory syncytial virus (RSV) infection [1]. Depending on age and subject comorbidities, RSV disease has a wide range of clinical manifestations, varying from a mild upper respiratory tract infection to a severe lower respiratory tract infection (LRTI) resulting in possible respiratory failure [1]. Preterm infants (defined as any infant born before the end of the 37th week of gestation [2,3]) are more prone to severe LRTI due to RSV disease, irrespective of the degree of prematurity [4,5]. Subsequently, these infants experience a more complicated clinical course compared with term infants, as demonstrated by increased length of hospital stay and a greater use of supplemental oxygen and respiratory support, and an increased risk for mortality [6–8].

Globally, an estimated 15 million infants are born prematurely every year [9]. As the largest proportion of premature births occur between 32 and 37 weeks of gestation [9], risk assessment of morbidity and mortality in these neonates is of considerable importance. Despite the physiologic and developmental immaturity of preterm infants [10], the value of passive immunoprophylaxis against RSV disease for all preterm infants born between 33 and 35 weeks of gestation remains a topic of debate.

Several single-country epidemiologic studies have been conducted in infants born between 32 and 35 weeks in an attempt to define risk factors for severe RSV disease and develop predictive models to identify those preterm infants who would benefit the most from RSV immunoprophylaxis (Table 1) [11–17]. Results from these studies have provided reliable predictors of RSV hospitalization, and have been adopted in national clinical guidelines and reimbursement eligibility criteria [18–22]. However, global generalization of these risk factors and predictive models may be limited by regional biologic, socioeconomic, and environmental differences, all of which may influence the risk for severe RSV disease and subsequent hospitalization.

**Table 1:** Predictors for RSV Hospitalization in Infants Born at 32 to 36 Weeks Gestational Age Identified From Multivariable Analysis in Single-Country Studies.

Variable	FLIP2 [13]	PICNIC [15]	RISK [11]	LOLLIPOP [14]	Munich RSV Study [16,17]	REPORT [12]
N	5441	1832	2514	964	375	1642
RSV LRTI hospitalization, n (%)	202 (3.7)	66 (3.6)	129 (5.1)	38 (3.9)	20 (5.3)	57 (3.5)
GA, wk (+d)^a^	32(+1)–35(+0)	33(+0)–35(+6)	32(+1)–35(+6)	32–36	33–35	32–35
Country	Spain	Canada	Netherlands	Netherlands	Germany	United States
Design	Prospective control	Prospective control	Observational	Case control-comparative	Observational	Observational
Prognostic factor						
Chronologic age/date of birth^b^	X	X	X	ND		X
Gestational age 33 and 34 weeks				X	NS	ND
Tobacco smokers in home or exposure to passive smoke in the first year		X		X		X
Smoking during pregnancy	X			ND		
Siblings^c^	X^d^	X	X^d^	ND	X	X
SGA		X		NS		
Breastfeeding duration of <2 mo	NS		X	NS		
Family history of atopy			X			
Male sex		X		ND	X	
Day care attendance of infant	X^d^	X	X^d^	ND		NS

GA = gestational age; LRTI = lower respiratory tract infection; LOLLIPOP = Longitudinal Preterm Outcome Project; ND = not determined (multivariable); NS = not significant; PICNIC = Pediatric Investigators Collaborative Network on Infections in Canada; RSV = respiratory syncytial virus; SGA = small for gestational age.

^a^Days not reported for all studies.

^b^≤10 wk at the start of RSV season [13], born between 14 August and 1 December [11], or born in November, December, or January [15].

^c^School aged [13], preschool aged [12,15], aged <2 years [17], or any age [11].

^d^Siblings and day care attendance of infant analyzed together.

The present study was conducted in 23 culturally and regionally diverse countries across the northern temperate zone to identify a more universal set of risk factors associated with the development of severe RSV disease in preterm infants born between 33 and 35 weeks of gestation. This could serve as an evidence-based resource for targeted immunoprophylaxis and to evaluate the burden of RSV disease.

## Methods

### Study Design

The observational study, *P*redictors associated with RSV h*O*spitalization in *N*on-prophylaxed premature *I*nfants born between 33 weeks + 0 days and 35 weeks + 6 days of gestation (PONI) was conducted over 1 RSV season (1 October 2013 to 30 April 2014) at 72 participating study sites in 23 countries in Western Europe (Austria, France, Norway, Portugal, Sweden, and Switzerland), Eastern Europe (Bosnia, Bulgaria, Czech Republic, Estonia, Latvia, Lithuania, Slovakia, and Slovenia) and Russia, South Korea, Mexico, and the Middle East (Bahrain, Egypt, Jordan, Lebanon, Oman, and Saudi Arabia); all were sites that had similar RSV seasonality. Parents or legal guardians of infants fulfilling the inclusion criteria were offered the opportunity for their child to participate in the study.

### Inclusion/Exclusion Criteria

Sites that had the experience and ability to conduct clinical research and had an integration or link to the readmission hospital were eligible for study participation. Readmission hospitals were required to routinely conduct testing for RSV infection on admission of infants with respiratory diseases. Study sites were restricted to recruiting, as closely as possible, equal numbers of subjects by month of birth to be able to estimate the possible risk factor of age of the infant at the onset of the RSV season. Parents/legal guardians of each infant signed a subject authorization form (and gave written informed consent, where applicable). Preterm infants born between 33 weeks + 0 days and 35 weeks + 6 days of gestation who would be ≤6 months of age at 1 October 2013 or born between 1 April 2013 and 28 February 2014 (the birth-age defined recruitment period) were eligible for inclusion in the study. Preterm infants with bronchopulmonary dysplasia, other chronic lung disease, or hemodynamically significant congenital heart disease (hsCHD) were excluded from the study because guidelines used by many countries in the current study suggest/recommend passive immunoprophylaxis for infants with these conditions. Preterm infants with other underlying conditions, such as cystic fibrosis or Down syndrome, were not excluded; however, information regarding such diagnoses made during the study was collected at the end of the RSV season. Infants whose parent(s) planned to move from the study area or infants who had received or were planning to receive immunoprophylaxis for severe RSV disease were excluded.

### Ethical Considerations

This epidemiologic study was approved by independent ethics committees **(S1 Table)** and/or independent review boards and run in compliance with local laws and regulations, with notification/submission to the responsible ethics committee, health institutions, and/or competent authorities as required by local laws and regulations and conducted according to the principles expressed in the Declaration of Helsinki. To maintain subject confidentiality, no demographic data that could identify the subject were collected (eg, initials or date of birth), and a unique number was assigned to each subject.

### Data Collection

Baseline (birth) data of the recruited infants were collected from birth records and each infant’s parents/guardians, who were contacted directly before discharge or by telephone after discharge. Data included subject selection criteria, infant characteristics (gestational age, birth weight, etc), perinatal history, and parental and family demographics. After the end of the RSV season (defined as 30 April 2014 for this study), all parents or legal guardians of the included infants were contacted by phone. During this call, baseline data were verified and information regarding whether the infant was hospitalized for LRTI was collected. Hospitalization decisions were made by the individual physicians based on clinical experience and local practice. For infants hospitalized because of LRTI, the admitting hospital site study coordinator completed an additional form using the subject’s hospitalization record, including rating of the respiratory illness severity as mild, moderate, or severe, by the opinion of the investigator (there was no scoring system available that would be familiar to all participating clinicians). LRTI infections associated with RSV were confirmed by the admitting hospital using standard RSV testing methods (rapid antigen, direct fluorescent antibody, polymerase chain reaction, immunofluorescence, or other assays). If an infant was hospitalized more than once for a confirmed RSV-positive LRTI, the hospitalization with the worst severity (as defined by the supervising physician) was used as the RSV hospitalization for the data analysis.

### Endpoints

The primary endpoint of the PONI study was hospitalization due to laboratory-confirmed RSV LRTI during the prevailing RSV season (defined as 1 October 2013 to 30 April 2014). The primary objective of the PONI study was to derive predictive factors (risk factors) for RSV LRTI hospitalization, based on the observation of whether a predictive factor was present or absent in controls (ie, infants without an RSV LRTI hospitalization) compared with cases (ie, infants with an RSV LRTI hospitalization). More precisely, predictive factors were evaluated for infants that were hospitalized because of LRTI with at least one positive RSV test. For this target, the reference group was infants that were not hospitalized because of LRTI. The data points collected were based on information that would be available at infant discharge and by previous studies in cohorts of infants born between 33 and 35 weeks of gestation [11,13,15]. Secondary endpoints included the incidence, severity, course, and outcomes of hospitalizations (RSV LRTI and non-RSV LRTI).

### Statistical Analyses

Standard descriptive statistics were used to describe the data; counts and percentages for categorical data and mean and standard deviation for continuous data are provided. In the analysis of total events, subjects with multiple events were counted once (eg, a subject with 2 RSV hospitalizations was counted once). The observation period for each infant was variable; therefore, incidence rates of RSV LRTI hospitalization were also calculated based on the cumulative time of observation for the infants observed. To enable comparison with other studies [15,23,24], rates were normalized per 100 infant years both for the whole study period (September 2013–July 2014) and for the RSV season (October 2013–April 2014). Only hospitalizations and person time contributed during these specified times were included. For comparison of infants with RSV-positive versus infants with RSV-negative tested LRTI hospitalization *P* values were derived using the chi-squared test for qualitative variables and the Wilcoxon rank sum test for quantitative variables.

For the derivation of a prediction rule, the following procedure was applied. First, bivariate associations were examined that describe the relationship of each potential predictive factor with the outcome of RSV LRTI hospitalization, reporting odds ratios (OR) from logistic regression analysis with corresponding two-sided Wald 95% confidence intervals (CIs) and corresponding *P* values. A level of 0.05 was considered significant for bivariate analysis.

Second, multivariable logistic regression models were used to investigate the combined influence of potential predictive factors and to find a comprehensive, yet parsimonious, predictive model with optimal predictive performance. To this end, two different variable selection procedures were applied to the set of predictive factors identified by the bivariate association analyses. A backward selection procedure was applied to selected factors (regardless of significance level identified in the bivariate analyses). At each backward selection step, a 5-fold cross-validation was used to determine the average predictive performance, which was quantified using the area under the receiver operating characteristic curve (AUROC). The set of variables was chosen that yielded the maximal average AUROC estimate across all backward selection steps [25]. To evaluate multivariable methods, optimism-corrected performance values, that is, the differences between the area under the curve (AUC) of the bootstrap and the AUC of the original data [25], were calculated using 250 bootstrap samples. Model discrimination was quantified using the AUROC and optimism-corrected performance; practical considerations were also used to derive a predictive model that would be easy to implement in clinical practice. All statistical analyses were carried out using SAS^®^ 9.2 (SAS Institute Inc., Cary, NC).

### Sample size

The study plan was to include a total of 2000 infants. Based on an assumed overall RSV LRTI hospitalization rate in infants that did not receive prophylaxis of approximately 5%, approximately 100 subjects were expected to be hospitalized for RSV-related LRTI.

For prognostic factors approximately equally distributed in the population (eg, sex), an overall RSV hospitalization rate difference of at least 4% (in any direction) would have a statistical power of 90% or higher. Incidence rates for prognostic factors between 10% and 50% in the study population and assumed RSV infection hospitalization rates for subjects with the respective factor of either 7% or 8% would provide at least 80% power in a number of different potential scenarios and outcomes.

## Results

### Study Population

**Fig 1:**
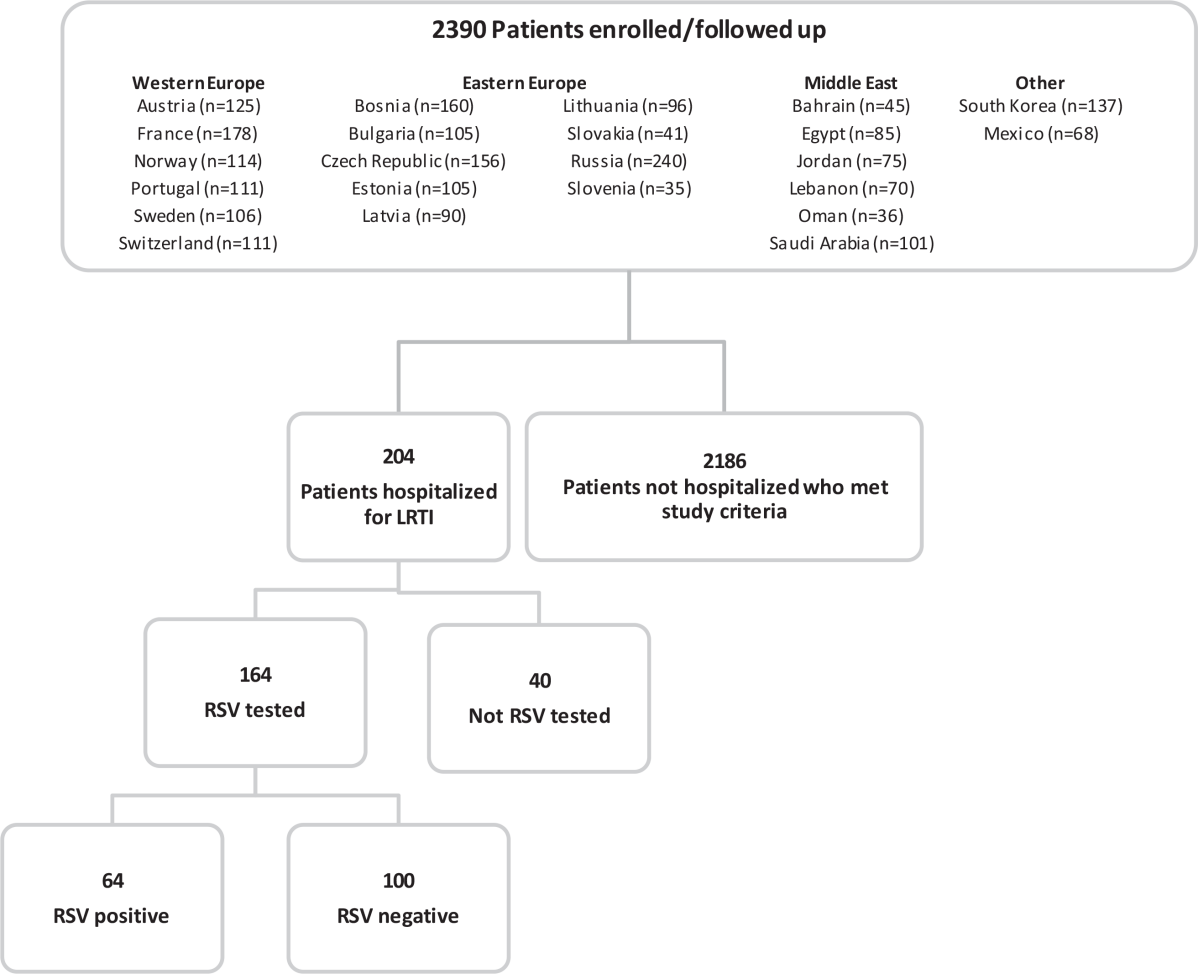
Study Flow Diagram. LRTI = lower respiratory tract infection; RSV = respiratory syncytial virus.

A total of 2390 infants (52.5% male) met the study criteria and were included in the analysis **(Fig 1)**. Subjects were enrolled at 72 sites across 23 countries. Russia had the highest enrollment with 10% of subjects enrolled. The number of infants born in each of the months before the onset of and during the RSV season was evenly balanced, with 7% to 10% of subjects born at each month from April 2013 through February 2014. The sociodemographic information at baseline for the infants is provided in **Table 2**. Overall, 1285 infants were born before 1 October 2013 (start of the RSV season). For those infants, the mean age at start of the RSV season was 3.0 months (range, 0.5–5.6 months). For the 1105 infants born after 1 October 2013 (ie, during the RSV season), the mean duration to their birthday was 2.4 months (range, 0.5–4.5 months). The study population consisted of approximately equal proportions of infants born by weeks of gestational age (wGA). The respective numbers of infants born at 33 wGA, 34 wGA, and 35 wGA were 26.3% (629/2390), 36.5% (873/2390), and 37.2% (888/2390). The majority of the infants (77.2%; 1846/2390) were white and more than a third (36.2%; 866/2390) of the births were multiples. Factors that may contribute to the multiple pregnancy rate noted in this study include the number of infants conceived by assisted fertilization (18.1%; 432/2390) and number of mothers who were more than 30 years of age at the time of delivery (52.5%; 1254/2390). Most infants (1479/2383; 61.9%) were born via cesarean delivery. Approximately three-quarters of the infants (73.1%) were breastfed for any period of time and 57.6% (1004/1742) of the breastfed infants were fed breast milk for ≥3 months. More than one-third of the infants (35.4%) lived in a household with a smoker. Approximately half of the infants (49.7%) had a comorbidity; the most common comorbidities included neonatal jaundice (446/2390; 18.7%) and respiratory distress syndrome (391/2390; 16.4%).

**Table 2:** Baseline Demographics and Characteristics in the Total Study Population and in Subjects Hospitalized for LRTI and Tested for RSV During Study Period.

Variable	Total (N = 2390)	Hospitalized for LRTI		*P* value^a^
		RSV Positive (n = 64)	RSV Negative (n = 100)	
*Infant and perinatal history*				
Male, n (%)	1255 (52.5)	37 (57.8)	63 (63.0)	0.51
Geographic region, n (%)				0.08
Eastern Europe and Russia	1028 (43.0)	14 (21.9)	39 (39.0)	
Middle East	412 (17.2)	27 (42.2)	37 (37.0)	
Western Europe	745 (31.2)	23 (35.9)	23 (23.0)	
Other	205 (8.6)	0	1 (1.0)	
Race, n (%)				0.31
White	1846 (77.2)	58 (90.6)	82 (82.0)	
Black	42 (1.8)	0	2 (2.0)	
Asian	161 (6.7)	0	2 (2.0)	
Mixed/Other	337 (14.1)	6 (9.4)	14 (14.0)	
Missing	4 (0.2)	0	0	
Multiple-birth infants, n (%)	866 (36.2)	16 (25.0)	38 (38.0)	0.08
Gestational age, wk, n (%)				0.03
33	629 (26.3)	8 (12.5)	30 (30.0)	
34	873 (36.5)	27 (42.2)	35 (35.0)	
35	888 (37.2)	29 (45.3)	35 (35.0)	
Infants born before 1 October 2013,^b^ n (%)	1285 (53.8)	33 (51.6)	60 (60)	0.29
Infants born on or after 1 October 2013,^b^ n(%)	1105 (46.2)	31 (48.4)	40 (40)	
Mean (SD) birth weight, g	2173.2 (433.1)^c^	2238.4 (470.6)	2163.5 (499.5)	0.24
Small for GA,^d^ n (%)	345 (14.4)	11 (17.2)	25 (25.0)	0.49
Average for GA,^d^ n (%)	1961 (82.1)	49 (76.6)	70 (70.0)	
Large for GA,^d^ n (%)	82 (3.4)	4 (6.3)	5 (5.0)	
Missing, n (%)	2 (0.1)	0	0	
Delivery, n (%)				0.19
Vaginal	904 (37.8)	29 (45.3)	35 (35.0)	
Cesarean	1479 (61.9)	35 (54.7)	65 (65.0)	
Missing	7 (0.3)	0	0	
Comorbidities, n (%)				
Any	1189 (49.7)	25 (39.1)	41 (41.0)	0.81
Neonatal jaundice	446 (18.7)	7 (10.9)	7 (7.0)	0.38
Respiratory distress syndrome	391 (16.4)	5 (7.8)	5 (5.0)	0.46
Cystic fibrosis	10 (0.4)	0	1 (1.0)	0.42
Down syndrome	6 (0.3)	0	1 (1.0)	0.42
Assisted fertilization, n (%)	432 (18.1)	5 (7.8)	11 (11.0)	0.67
Initial hospital stay, n (%)				0.22
≥7 d	1892 (79.2)	42 (65.6)	69 (69.0)	
<7 d	461 (19.3)	20 (31.3)	21 (21.0)	
Missing	37 (1.5)	2 (3.1)	10 (10.0)	
*Maternal/family*				
Maternal age at delivery, y				0.72
Mean (SD)	30.8 (5.4)	29.1 (5.5)	29.3 (5.0)	
≤25, n (%)	329 (15.5)	21 (32.8)	21 (21.0)	0.09
>25, n (%)	1857(84.9)	43 (67.2)	79 (79.0)	
Education of mother,^e^ n (%)				0.54
Low	644 (26.9)	31 (48.4)	41 (41.0)	
Medium	1381 (57.8)	25 (39.1)	45 (45.0)	
High	330 (15.2)	6 (9.7)	13 (13.0)	
Missing	6 (0.3)	0	0	
Smoking during pregnancy, n (%)	207 (8.7)	12 (18.8)	19 (19.0)	0.92
Family history of atopy/allergy, n (%)				
Any family member	924 (38.7)	31 (48.4)	37 (37.0)	0.20
Maternal	536 (22.4)	13 (20.3)	22 (22.0)	0.69
Paternal	441 (18.5)	19 (29.7)	19 (19.0)	0.15
*Environmental characteristics*				
Breastfed, n (%)	1747 (73.1)	40 (62.5)	52 (52.0)	0.31
Smoker living with infant, n (%)	845 (35.4)	42 (65.6)	55 (55.0)	0.20
Household size >4, n (%)	369 (15.4)	13 (20.3)	18 (18.0)	0.74
Other children aged <18 y in household, n (%)	1317 (55.1)	37 (57.8)	61 (61.0)	0.68
Day care attendance, n (%)				
Infant	323 (13.5)	12 (18.8)	12 (12.0)	0.29
Other children in household	529 (22.1)	20 (31.3)	28 (28.0)	0.68

GA = gestational age; LRTI = lower respiratory tract infection; RSV = respiratory syncytial virus.

^a^*P* value is for RSV positive vs RSV negative.

^b^Defined as onset of the RSV season.

^c^n = 2389.

^d^Defined per Fenton and Kim [26].

^e^Low = no formal education, primary school or secondary school; medium = professional non-university or university (14–18 years of education); high = higher education >18 years.

Rates of RSV-related hospitalization are outlined in **Table 3**. The normalized RSV hospitalization rates (per 100 infant years) for the whole study period (September 2013–July 2014) and for the RSV season (October 2013–April 2014) were 4.1% and 6.1%, respectively.

**Table 3:** RSV Hospitalization Rates per 100 Infant Years.*

**Hospitalization during study period**								
	LRTI		LRTI RSV positive		LRTI RSV negative		LRTI RSV unknown	
Number of infant years	Number of subjects	Incidence per 100 infant years	Number of subjects	Incidence per 100 infant years	Number of subjects	Incidence per 100 infant years	Number of subjects	Incidence per 100 infant years
1547.0	204	13.2	64	4.1	105	6.8	51	3.3
**Hospitalization during RSV season**								
	LRTI		LRTI RSV positive		LRTI RSV negative		LRTI RSV unknown	
Number of infant years	Number of subjects	Incidence per 100 infant years	Number of subjects	Incidence per 100 infant years	Number of subjects	Incidence per 100 infant years	Number of subjects	Incidence per 100 infant years
756.5	127	16.8	46	6.1	58	7.7	32	4.2

LRTI = lower respiratory tract infection; RSV = respiratory syncytial virus.

*An infant with >1 hospitalization could contribute to multiple event types (ie, LRTI hospitalization with positive RSV test, LRTI hospitalization with negative RSV test, LRTI hospitalization with unknown RSV test). However, subjects within a type were counted only once (eg, a subject with 2 hospitalizations with negative RSV test was counted once).

### Predictors for RSV Hospitalization

The association between RSV LRTI hospitalizations (over the entire study period) and each dichotomized potential predictive factor was analyzed on the basis of bivariate frequency distributions. The ORs and corresponding 95% confidence limits (CLs) for factors which appeared to have a significant relationship with RSV LRTI hospitalization (based on significance level of 0.05) is reported **(Table 4)**.

**Table 4:** Statistically Significant (*P*<0.05) Bivariate Associations for Predictive Factors for RSV Hospitalization.

Predictor	Hospitalized/ Not Hospitalized With Risk Factor Present, n/N (%)	Hospitalized/ Not Hospitalized With Risk Factor Absent, n/N (%)	*P* Value	OR Estimate (95% CI)
*Infant and perinatal history*				
Gestational age >33 wk (vs <33 wk)	56/1610 (3.5)	8/576 (1.4)	0.0161	2.50 (1.19–5.28)
Non-hsCHD diagnosis	5/51 (9.8)	59/2135 (2.8)	0.0093	3.55 (1.37–9.22)
Initial hospital stay <7 d (vs ≥7 d)	20/413 (4.8)	42/1749 (2.4)	0.0117	2.01 (1.17–3.46)
*Maternal/Paternal*				
Maternal smoking during pregnancy (yes vs no)	12/175 (6.9)	46/1944 (2.4)	0.0014	2.90 (1.51–5.58)
Age of mother at delivery ≤25 y (vs >25 y)	21/329 (6.4)	43/1857 (2.3)	0.0002	2.76 (1.62–4.71)
Low educational level of mother (vs high or medium)	31/560 (5.5)	33/1620 (2.0)	<0.0001	2.89 (1.74–4.80)
Atopy of father (yes vs no)	19/396 (4.8)	37/1578 (2.3)	0.0128	2.05 (1.17–3.60)
*Environment*				
Not breastfed (vs breastfed)	23/510 (4.5)	40/1627 (2.5)	0.0227	1.84 (1.09–3.10)
Smoking of family members (yes vs no)	42/722 (5.8)	22/1425 (1.5)	<0.0001	3.77 (2.23–6.37)
Children aged 4–5 y present (yes vs no)	22/358 (6.1)	42/1828 (2.3)	0.0003	2.68 (1.58–4.54)

non-hsCHD = non-hemodynamically significant congenital heart disease; OR = odds ratio; RSV = respiratory syncytial virus.

**Fig 2:**
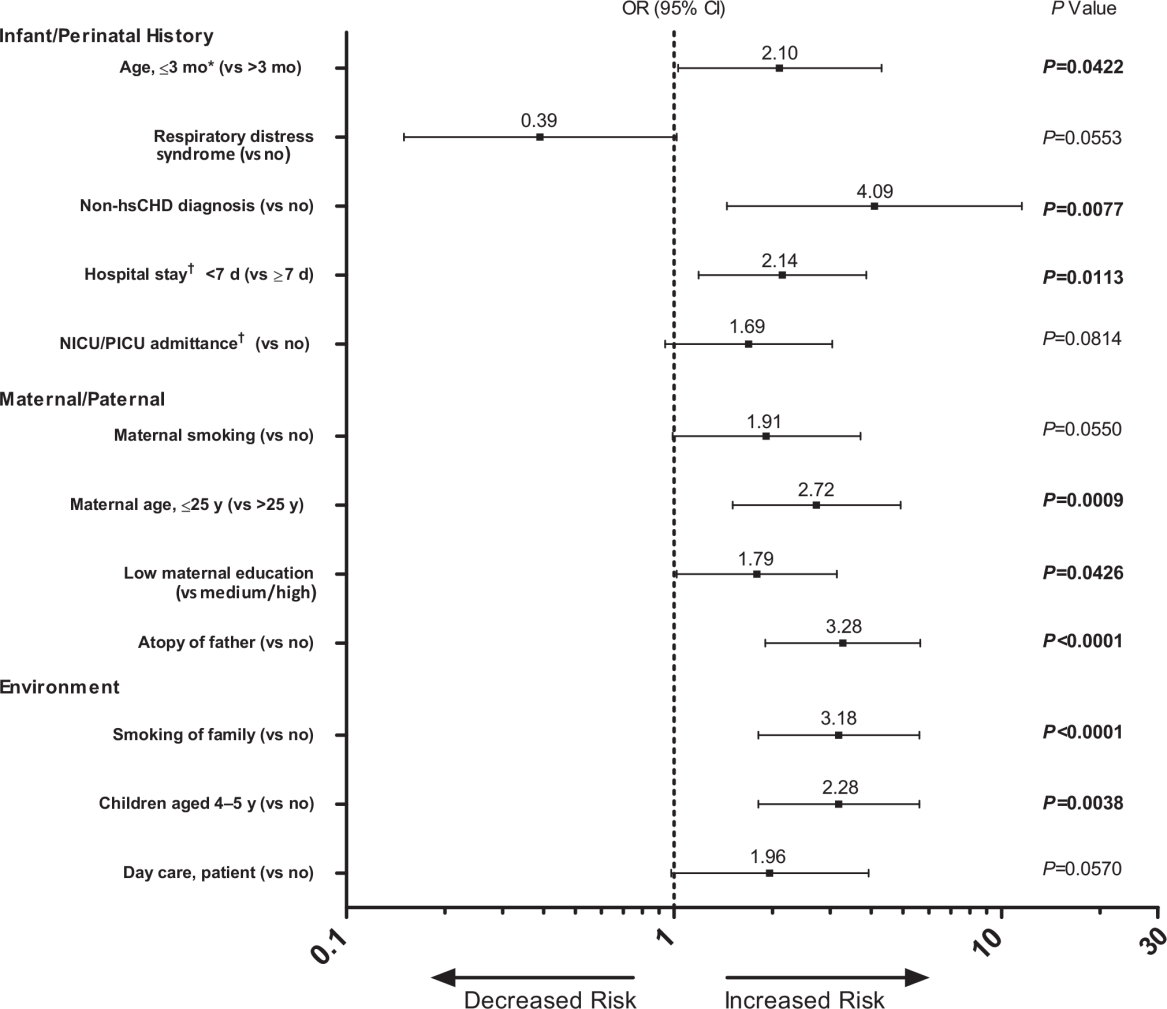
Odd Ratios of Risk Factors for RSV-Associated Hospitalization in Multivariable Analysis (Selected by Backward Selection). ICU/PICU = neonatal/pediatric intensive care units; non-hsCHD = non-hemodynamically significant congenital heart disease; OR = odds ratio; RSV = respiratory syncytial virus. *On 1 October. ^†^At birth.

For the multivariable analyses, 36 factors, regardless of the associated significance level in the bivariate analyses, were considered and a 12-variable model was selected by backward selection **(Fig 2)**. In this multivariable logistic regression analysis, the following factors were statistically significant: smoking of family members (*P*<0.0001), non-hsCHD diagnosis (*P* = 0.0077), maternal age of ≤25 years at delivery (*P* = 0.0009), low educational level of the mother (*P* = 0.0426), presence of children aged 4 to 5 years in the household (*P* = 0.0038), age ≤3 months on 1 October (*P* = 0.0422), and presence of atopy in the father (*P*<0.0001).

Because a 12-variable predictive model for RSV LRTI hospitalization may be difficult to implement in daily medical practice, models of 4 and 6 predictive factors were selected based on the number of missing values and practical availability. The highest missing frequency was observed with the following factors: atopy of father (8/64 missing values; 12.5%) and smoking of the mother during pregnancy (6/64 missing values; 9.4%).

Based on bivariate OR estimates, the lack of missing data, and practical availability, a total of 6 models of 4 and 6 predictive factors each were selected for performance estimation analysis **(Table 5)**. Models A and C were determined to have the best accuracy for the prediction of RSV LRTI-related hospitalization based on the respective optimism-corrected performance scores of 0.753 and 0.755. Common prognostic factors in both models were age ≤3 months on 1 October, smoking of family members, maternal age ≤25 years at delivery, and the presence of children aged 4 to 5 years old in the household. Model A also included duration of initial hospital stay of <7 days and low educational level of the mother, whereas Model C included smoking of the mother during pregnancy and subject day care attendance.

**Table 5:** Predictive Models of 4 and 6 Prognostic Factors for Hospitalization Due to RSV-Associated LRTI With Optimism-Corrected Performance Value.

	6-Variable Models			4-Variable Models		
Model Number	A	B	C	D	E	F
Optimism-corrected performance value	0.753	0.735	0.755	0.733	0.740	0.655
Prognostic factor						
Age on 1 October ≤3 mo (vs >3 mo)	X	X	X	X		X
Smoking of family members (yes vs no)	X	X	X	X	X	
Age of mother at delivery ≤25 y (vs >25 y)	X	X	X	X	X	
Children 4–5 years old present (yes vs no)	X	X	X	X	X	X
Duration of initial hospital stay <7 d (vs ≥7 d)	X	X				
Low educational level of mother (vs medium or high)	X					
Non-hsCHD diagnosis (yes vs no)		X			X	
Smoking of mother during pregnancy (yes vs no)			X			X
Subject day care attendance (yes vs no)			X			X

LRTI = lower respiratory tract infection; non-hsCHD = non-hemodynamically significant congenital heart disease; RSV = respiratory syncytial virus.

### Hospitalization Outcomes

During the study period (September 2013–July 2014), 64 infants were hospitalized for RSV LRTI (4.1% incidence per 100 infant years). In more than half of these cases (35/64; 54.7%), the respiratory illness was subjectively rated as moderate by the investigator; nearly one third were rated as severe (19/64; 29.7%), and the respiratory illness was categorized as mild in 15.6% of the infants (10/64). A total of 73.4% of these subjects (47/64) required the use of supplemental oxygen, with a median (IQR) duration of 4 (3–7) days; 10.9% of subjects (7/64) required mechanical ventilation for a median (IQR) duration of 4 (3–7) days. Admission to the pediatric/neonatal intensive care unit (PICU/NICU) was reported in 19 of 64 infants (29.7%); the median (IQR) duration of the PICU/NICU stay was 6 (5–12) days.

## Discussion

Identification of the sociodemographic risk factors for severe RSV-associated LRTI and the implementation of targeted prophylaxis in vulnerable infants are important to reduce the burden of disease due to RSV. In the current study, data from culturally and regionally diverse countries were used to identify a simple yet predictive set of risk factors associated with the development of severe RSV disease in preterm infants between 33 and 35 weeks of gestation not receiving immunoprophylaxis.

The normalized RSV hospitalization rates (per 100 infant years) for the study period (September 2013–July 2014) and for the RSV season (October 2013–April 2014) were 4.1% and 6.1%, respectively. This observed incidence of RSV LRTI hospitalization is similar to the rates identified in another study of infants 32 to 35 wGA that utilized active surveillance for laboratory-confirmed RSV [12].

The PONI study was the first to use epidemiologic data from a multinational cohort of preterm infants born 33 to 35 wGA to identify predictors of hospitalization for RSV-associated LRTI. Findings suggest that a maternal age at delivery of ≤25 years was significantly associated with an increased risk of RSV-associated hospitalizations, which had not been previously identified as a risk factor in any of the other studies conducted in preterm infant populations of similar gestational age. Limited reports examining the relationship of maternal age and RSV-associated hospitalizations exist. In 1 report, an increased risk of hospitalization for RSV infection was associated with maternal age <25 years at delivery (compared with ≥25 years) in a multivariate analysis of a single-center retrospective study of children <2 years of age [27].

An association between lower levels of maternal education and severe RSV disease risk was also found in the PONI study. Few studies have reported relationships between parental educational levels and RSV disease risk. A significantly increased risk of hospitalization for RSV infection was noted for a maternal educational level of ≤12 years and for a paternal educational level of high school or less (ie, ≤12 years) in univariate analyses [15,28]. Conversely, a trend (non-statistically significant) for a decreased hospitalization risk with increased parental educational levels was noted in FLIP [17]. The increased risks associated with both younger maternal age and lower educational levels may be reflective of overall lower socioeconomic status, although the association of lower income/socioeconomic status with an increased risk of RSV infection remains controversial [29,30]. However, the relationship between socioeconomic status and morbidity is well known and may be attributed to multiple factors such as environment, health behaviors/access, or psychological state [29,31]. Assessments of socioeconomic status were not feasible in our study because of the complexity of such comparisons across countries/regions included.

The PONI study also confirmed risk factors for RSV-associated LRTI hospitalizations, such as living with smokers and the presence of other children/siblings (especially 4–5 years of age), that have been previously identified in studies using single-country Canadian, Western European, and American populations **(Table 1)** [11–17], suggesting that they are widely applicable as predictive factors. In addition, our model suggests that infants with non-hsCHD may also be at an increased risk of RSV-associated hospitalization, although it is well known that infants with hsCHD (who were excluded from this study) are at higher risk of severe RSV disease [32,33]. Importantly, the majority of factors associated with the increased risk of RSV infection identified in the current study can be assessed before postnatal discharge. Thus, strategies to reduce the risk of severe disease due to RSV infection (eg, immunoprophylaxis and limiting smoking and crowded areas) may be discussed with the parents of an infant before they leave the hospital.

A strength of the current study is that it uses data from a large cohort of infants from multiple northern hemisphere temperate zone countries to identify prognostic factors for RSV-related hospitalizations. Limitations include the observational study design and regional differences in the clinical suspicion of and diagnostic criteria for RSV infection, as well as testing protocols and methodologies for detecting RSV disease. National and regional differences in treatment practice and healthcare systems, especially the criteria for hospitalization, may have affected our overall risk factor findings and may also affect assessments of hsCHD (or non-hsCHD), disease severity, PICU admittance, and modes of ventilation. Notably, stringent disease severity classification criteria were not used and disease severity was subjectively based on individual investigators findings; limitations on data collected during hospitalizations precludes further analyses.

Although the 2 models presented that included non-hsCHD as a factor could be affected by the non-standardized definitions of non-hsCHD (ie, classified according to local guidelines/practice at the study sites) used in the study, we note that the 4 models presented that do not include it as a factor also show comparable performance. Approximately 20% of the infants identified as being hospitalized for LRTI in this study were not tested for RSV infection, which may have affected the number of subjects hospitalized with confirmed RSV LRTI. Because none of the patients who were diagnosed with cystic fibrosis or Down syndrome were determined to have a positive RSV infection and the total numbers were low, we do not believe that this was enough to confound the results of our analyses as presented. The true impact of some factors, such as maternal smoking or atopy of the father, may be underrepresented because of the relatively greater number of missing values for these variables in subject records. Site selection itself was inherently biased by the requirement for the experience and ability to conduct clinical research and integration or a link to the readmission hospital.

## Conclusions

Pediatricians and other healthcare workers should be aware that preterm infants born 33 to 35 wGA are at risk of RSV infection that could lead to hospitalization. The PONI study demonstrated a continued burden of disease from RSV disease over time with more current data, and identified risk factors associated with RSV hospitalization, such as maternal age and educational level, and confirmed other previously identified prognostic factors in a multinational population. Many of the risk factors associated with an increased risk of severe RSV-related LRTIs requiring hospitalization identified in this and previous studies appear to be environmental and thus can be addressed by educating parents on the need to limit or avoid infant exposure to certain situations (eg, crowded places or smoking) and the benefits of breastfeeding. The prognostic factors for RSV hospitalization found in the PONI study and the simple predictive model developed may be useful in clinical practice across a large number of geographic regions to identify infants who are at high risk for RSV-associated hospitalization for targeted intervention strategies, including immunoprophylaxis. Future studies should focus on universally applicable risk scoring tools (prediction models).

## Supporting Information

S1 Table Participating institutions and corresponding ethics committees/review boards. (PDF)

S1 Appendix Investigator Acknowledgments. (PDF)
