# Supplementary material for: Predictors of RSV LRTI Hospitalization in Infants Born at 33 to 35 Weeks Gestational Age: A Large Multinational Study (PONI)
Source: PLoS One. 2016 Jun 16;11(6):e0157446. doi: 10.1371/journal.pone.0157446 (PMC4910988; doi:10.1371/journal.pone.0157446)
Supplement: S1 Table — (PDF) [file pone.0157446.s001.pdf]

S1 Table. Participating institutions and corresponding ethics committees/review boards

Institution	Ethics Committee/Review Board
University Clinic for Pediatric Medicine, General Hospital Vienna - Medical University Vienna	Ethikkommission der Medizinischen Universität Wien
University Clinic for Pediatric Medicine, Medical University Graz	Ethikkommission der Medizinischen Universität Graz
Department for Pediatric Medicine, Medical University Innsbruck	Ethikkommission der Medizinischen Universität Innsbruck
University Clinic for Pediatric Medicine, Paracelsus Medical University	Ethikkommission für das Bundesland Salzburg
Salmaniya Medical Complex	Office of Assistant Undersecretary for Training and Planning
University Hospital Clinical Center Banja Luka	Ethics Committee of the University Hospital Clinical Center Banja Luka
University Clinical Center Tuzla	Ethics Committee of the University Clinical Center Tuzla
University Clinical Hospital Mostar	Ethics Committee of the University Clinical Hospital Mostar
Clinical University Center Sarajevo	Ethics Committee of the University Clinical Center Sarajevo
UMBAL Georgi Stranski	Ethics Committee UMBAL Georgi Stranski
SBALAG Maichin Dom	Ethics Committee SBALAG Maichin Dom
SBALDB EAD	Ethics Committee SBALDB EAD
UMBAL St. Kirkovitch	Ethics Committee UMBAL St. Kirkovitch
UMBAL Sveti Georgi EAD	Ethics Committee UMBAL Sv Georgi EAD
The Institute for the Care of Mother and Child, Neonatological Ward	Etická Komise, ÚPMD
Pilsen Faculty Hospital, Edvarda Beneše	Ethics Committee, tř. Dr.E.Beneše
Ain Shams University Faculty of Medicine Pediatric Department	Ethical Committee of Ain Shams University
Cairo University Faculty of Medicine Pediatric Department	Ethical Council of Pediatric Department Cairo University
Tartu University Hospital	Tallinn Medical Research Ethics Committee
West Tallinn Central Hospital	Tallinn Medical Research Ethics Committee
East Tallinn Central Hospital	Tallinn Medical Research Ethics Committee
Hôpital Clocheville	Comité consultatif sur le traitement de l'information en matière de recherche dans le domaine de la santé (CCTIRS)
Hôpital Charles Nicolle	Comité consultatif sur le traitement de l'information en matière de recherche dans le domaine de la santé (CCTIRS)

CHU Bocage	Comité consultatif sur le traitement de l'information en matière de recherche dans le domaine de la santé (CCTIRS)
Hôpital Arnaud De Villeneuve	Comité consultatif sur le traitement de l'information en matière de recherche dans le domaine de la santé (CCTIRS)
Hôpital De La Conception	Comité consultatif sur le traitement de l'information en matière de recherche dans le domaine de la santé (CCTIRS)
King Abdullah University Hospital	King Abdullah University Hospital
University Children's Hospital, Riga	Riga East Clinical University Hospital Support Foundation's Medical and Biomedical Research Ethics Committee
Hotel-Dieu de France	Comite d'ethique - Universite Saint Joseph
Hammoud Hospital - University Medical Center	Hammoud Hospital UMC Institutional review Board
Makassed General Hospital	Makassed General Hospital Institutional Review Board
American University of Beirut	American University of Beirut Institutional Review Board
Children's Hospital, Affiliate of Vilnius University Hospital Santariskiu Klinikos	Lithuanian Bioethics Committee
Hospital of Lithunian University of Health Sciences, Kaunas Clinics	Lithuanian Bioethics Committee
Hospital General Regional de León la SSA	Comité de Ética en Investigación, Hospital León
Hospital Metropolitano "Dr. Bernardo Sepúlveda"	Comité de Ética en Investigación, Hospital Metropolitano Dr. Bernardo Sepúlveda
Akershus University Hospital	REC North; Regional Committees for Medical and Health Research Ethics
St. Olavs University Hospital	REC North; Regional Committees for Medical and Health Research Ethics
Stavanger University Hospital	REC North; Regional Committees for Medical and Health Research Ethics
Oslo University Hospital Rikshospitalet	REC North; Regional Committees for Medical and Health Research Ethics
Oslo University Hospital Ullevål	REC North; Regional Committees for Medical and Health Research Ethics
Sultan Qaboos University Hospital	Sultan Qaboos University Ethics Committee
Hospital Prof. Doutor Fernando Fonseca, E.P.E.	Comissão de Ética do Hospital Prof. Fernando Fonseca , E.P.E.
Centro Hospitalar São João	Comissão de Ética do Hospital de São João
Hospital de S. Francisco Xavier - CHLO, E.P.E.	Comissão de Ética do Centro Hospitalar de Lisboa Ocidental, E.P.E - Hospital Egas Moniz

GUZ "Clinical Hospital #5"	Independent Interdisciplinary Committee for Ethical Review of Clinical Studies
MAUZ of Irkutsk GIMDKB. OPNND	Independent Interdisciplinary Committee for Ethical Review of Clinical Studies
GBUZ City Children Hospital #1 MZ RT	Independent Interdisciplinary Committee for Ethical Review of Clinical Studies
FGBU "NTSZD" RAMS	Independent Interdisciplinary Committee for Ethical Review of Clinical Studies
Pediatric Infectious Clinical Hospital #6	Independent Interdisciplinary Committee for Ethical Review of Clinical Studies
Moscow Research Institute of Pediatrics and Pediatric Surgery - Correction Center of Premature Infants	Independent Interdisciplinary Committee for Ethical Review of Clinical Studies
FGBU NC AGiP n.a. V.I. Kulakova Minzdrava RF	Independent Interdisciplinary Committee for Ethical Review of Clinical Studies
Municipal Budget Health Care Institution of Novosibirsk "City Perinatal Center"	Independent Interdisciplinary Committee for Ethical Review of Clinical Studies
Correction Center of Samara	Independent Interdisciplinary Committee for Ethical Review of Clinical Studies
Children Hospital #17	Independent Interdisciplinary Committee for Ethical Review of Clinical Studies
LPU GUZ Perinatal Center of Saratov	Independent Interdisciplinary Committee for Ethical Review of Clinical Studies
Independent Regional State Health Care Institution Tomsk Regional Perinatal Center	Independent Interdisciplinary Committee for Ethical Review of Clinical Studies
BU "Presidential Perinatal Center"	Independent Interdisciplinary Committee for Ethical Review of Clinical Studies
MBUZ Children Hospital #8	Independent Interdisciplinary Committee for Ethical Review of Clinical Studies
State Educational Institution of Higher Professional Education "Chita State Medical Academy" of the Ministry of Health Care of Russian Federation	Independent Interdisciplinary Committee for Ethical Review of Clinical Studies
King Abdulaziz Medical City National Guard Hospital	KING ABDULLAH INTERNATIONAL MEDICAL RESEARCH CENTER, King Abdul-Aziz , Medical City, Ministry of National Guard Health Affairs
King Fahd Medical City	King Fahd Medical City
Martin University Hospital	Etická komisia Univerzitnej nemocnice Martin
Hospital for Gynecology and Obstetrics Postojna	Republic of Slovenia National Medical Ethics Committee (NMEC)
Hospital for Gynecology and Obstetrics Kranj	Republic of Slovenia National Medical Ethics Committee (NMEC)

Samsung Medical Center	Samsung Medical Center Institutional Review Board
Seoul National University Hospital	Seoul National University Hospital Institutional Review Board
Asan Medical Center	Asan Medical Center Institional Review Board
Neonatology Department, Astrid Lindgren's Hospital, Danderyd, Karolinska University Hospital	Neonatology Department, Astrid Lindgren's Hospital, Danderyd, Karolinska University Hospital
Neonatal Intensive Care Unit, Linköping University Hospital	Neonatal Intensive Care Unit, Linköping University Hospital
Sachs Children and Youth Hospital	Sachs Children and Youth Hospital
Division of Infectious Diseases and Hospital Epidemiology, and Children’s Research Center, University Children’s Hospital Zurich, University of Zurich	Kantonale Ethikkommission Zürich
University of Basel Children's Hospital	Ethikkommission Nordwest- und Zentralschweiz (EKNZ)
Children’s Hospital of Geneva, Department of Pediatrics, University Hospitals of Geneva & Geneva Medical School	Commission cantonale d'éthique de la recherche (CCER)
