# Supplementary material for: Predictors of RSV LRTI Hospitalization in Infants Born at 33 to 35 Weeks Gestational Age: A Large Multinational Study (PONI)
Source: PLoS One. 2016 Jun 16;11(6):e0157446. doi: 10.1371/journal.pone.0157446 (PMC4910988; doi:10.1371/journal.pone.0157446)
Supplement: S1 Appendix — (PDF) [file pone.0157446.s002.pdf]

S1 Appendix. Investigator Acknowledgments

We would like to acknowledge the investigators who participated in this study:

Austria: Angelika Berger, Ursula Kiechl-Kohlendorfer, Bernhard Resch, Martin Wald

Bahrain: Ali Ebrahim

Bosnia and Herzegovina: Suada Heljić, Hajrija Maksić, Veroslava Milošević, Fahrija Skokić, Darinka Šumanović-Glamuzina

Bulgaria: Victoria Atanasova, Ralitza Georgieva, Maya Krasteva, Hristo Mumdzhiev, Boryana Slantcheva

Czech Republic: Jiri Dort, Zbynek Stranak

Egypt: Hisham Awad, Moataza Bachir, Nahed Fahmy, Safaa Shafik

Estonia: Pille Andresson, Eha Kallas, Pille Saik

France: Gilles Cambonie, Patricia Garcia, Maria Grzegorzewicz, Didier Pinquier, Elie Saliba, Denis Semama

Jordan: Wadah Khriesat

Latvia: Daiga Kviluna

Lebanon: Ghassan Baasiri, Imad Melki, Mariam Rajab, Khalid Yunis

Lithuania: Nijole Drazdiene, Rasa Tameliene

Mexico: Amalia Becerra Aquino, Jairo Barajas Rangel

Norway: Kristin Brække, Therese Farstad, Knut Øymar, Kari Risnes, Arild Rønnestad

Oman: Hussein Al Kindi

Portugal: Maria Helena Carreiro, Hercília Guimarães, António Macedo

Russia: Irina Vladimirovna Davydova, Elena Aleksandrovna Degtiareva, Valery Viktorovich Gorev, Elena Solomonovna Keshishyan, Svetlana Nikolaevna Kuznetsova, Alexandra Sergeevna Panchenko, Larisa Evgenievna Prokopenko, Olga Gennadievna Reutskaya, Konstantin Vladislavovich Romanenko, Tatyana Aleksandrovna Romanova, Irina Ivanovna Ryumina, Asiya Ildusovna Safina, Tatyana Yurievna Sverschevskaya, Irina Valerievna Vinogradova, Tatyana Evgenievna Zayachnikova

Saudi Arabia: Fahad Alaql, Saif Al Saif

Slovakia: Mirko Zibolen

Slovenia: Petra Bratina, Andreja Cerkvenik-Škafar

South Korea: Yun Sil Chang, AR Kim, Han-Suk Kim

Sweden: Eva Berggren Broström, Paraskevi Kosma, Fredrik Lundberg

Switzerland: Ulrich Heininger, David Nadal, Klara Posfay-Barbe
